# Consumption of whole grains in French children, adolescents and adults

**DOI:** 10.1017/S0007114514002670

**Published:** 2014-10-10

**Authors:** France Bellisle, Pascale Hébel, Justine Colin, Béatrice Reyé, Sinead Hopkins

**Affiliations:** 1 Nutritional Epidemiology Research Unit, UMR U557 INSERM, U1125 INRA, CNAM, Paris 13 University, CRNH-IdF, Bobigny, France; 2 CREDOC (Centre de Recherche pour l'Etude et l'Observation des Conditions de Vie), 142 rue du Chevaleret, Paris, France; 3 Cereal Partners Worldwide, Lausanne, Switzerland

**Keywords:** Whole grains, Dietary surveys, French population

## Abstract

The consumption of whole grain foods is associated with many nutritional, health and weight control benefits. The present study assessed whole grain intake in France on the basis of a 7 d dietary survey in a representative sample of children, adolescents and adults (Comportements et Consommations Alimentaires en France 2010 survey). Special care was taken to identify and assess the intake of all whole grains. All foods consumed were considered, with no lower limit on whole grain content. For the majority of foods, details regarding the whole grain contents were obtained from brand information and quantitative nutrient declarations on food labels. Over half of the respondents reported never consuming any whole grain. In participants who did, consumption levels were very low (about 9·1 g/d in children and 14·4 g/d in adults). The main food sources of whole grains were breakfast cereals in children and adolescents and bread in adults. Consumers of whole grains had higher daily intakes of fibre and several vitamins and minerals than non-consumers. In adults but not in children, the OR for overweight/obesity decreased significantly as the level of whole grain consumption increased. Although a majority of French consumers comply with the national recommendation to consume a starchy food with each meal, they do so with minimal consumption of whole grain foods.

Epidemiological evidence has consistently demonstrated an association between whole grain consumption and various health benefits including a reduced risk of type 2 diabetes^(^ ^1^ ^,^ ^2^ ^)^ and CVD^(^ ^1^ ^,^ ^3^ ^)^ and a lower body weight^(^ ^4^ ^–^ ^6^ ^)^. Findings from short-term intervention studies have been less conclusive, with some studies reporting beneficial effects of whole grain consumption on cholesterol concentrations^(^ ^7^ ^)^, blood pressure^(^ ^8^ ^)^ and insulin sensitivity^(^ ^9^ ^)^, while others reported no significant beneficial effects on these health outcomes^(^ ^10^ ^,^ ^11^ ^)^. A 2013 systematic review and meta-analysis of twenty-six randomised controlled trials reported no effect of increasing whole grain consumption on body weight, but a small significant effect on fat mass^(^ ^12^ ^)^.

According to the American Association of Cereal Chemists International (AACCI), whole grains ‘consist of the intact, ground, cracked or flaked caryopsis, whose principal anatomical components – the starchy endosperm, germ and bran – are present in the same relative proportions as they exist in the intact caryopsis’^(^ ^13^ ^)^. In 2014, the HEALTHGRAIN forum, an organisation arising from the European Union-funded HEALTHGRAIN project^(^ ^14^ ^)^, completed the AACCI definition to reflect the current industrial practices for production of flours and consumer products. Whole grain foods and ingredients are recognised to have a superior nutrition composition compared with their refined counterparts owing to higher levels of fibre, some vitamins (vitamin E and B vitamins), minerals (Fe, P, Mg and Zn) and phytochemicals^(^ ^15^ ^)^. Despite the clear definition of ‘whole grains’, there is no international consensus on what constitutes a ‘whole grain food’ and definitions vary widely between countries. Recently, however, a multidisciplinary panel of American and European experts^(^ ^16^ ^)^ has proposed that a food providing at least 8 g of whole grains/30 g serving be defined as a ‘whole grain food’ for purposes of dietary recommendations.

The WHO recommends an increase of whole grain consumption as a strategy to reduce the risk of obesity, CVD and diabetes^(^ ^17^ ^)^. Furthermore, whole grain consumption is recommended by public health authorities in several countries with recommendations varying in terms of their qualitative and quantitative descriptions^(^ ^18^ ^)^. For example, a quantitative recommendation exists in Denmark whereby at least 75 g of whole grains are recommended in a 10 MJ (2400 kcal) daily diet through the consumption of whole grain breakfast products, bread, rice and pasta^(^ ^19^ ^)^. In the USA, three 16 g servings of whole grains are recommended daily (48 g total) representing at least half of the total recommended grain servings^(^ ^20^ ^)^. The Canadian Food Guide recommends three to eight servings of grain products daily, with at least half of the servings from whole grains^(^ ^21^ ^)^. In other countries such as the UK and Germany, the dietary guidelines simply encourage consuming whole grain varieties of grain products^(^ ^22^ ^)^. In spite of these recommendations, the mean daily intake of whole grains is generally low in Europe and America, ranging from 10 to 20 g in children^(^ ^23^ ^–^ ^27^ ^)^ and from 10 to 60 g in adults^(^ ^28^ ^–^ ^30^ ^)^.

In France, the National Programme on Nutrition and Health (PNNS) recommends the consumption of at least one starchy product at every meal occasion, in variable amounts according to the consumer's appetite^(^ ^31^ ^)^. ‘Starchy foods’ include potatoes and legumes as well as cereal products, and although consumers are encouraged to consume ‘whole foods’, one could comply with the PNNS recommendations without consuming any whole grain food. Previous efforts to assess whole grain consumption in the French population have focused only on the number of servings of whole grain foods, without specifically quantifying absolute whole grain intake or differentiating whole grain sources^(^ ^32^ ^–^ ^34^ ^)^.

Therefore, the aims of the present study were to estimate whole grain intake from all dietary sources in French children and adults and to compare it with recommendations. In addition, the associations between whole grain consumption, daily nutrient intakes and body weight status were examined. The intake data were obtained from the Comportements et Consommations Alimentaires en France (CCAF) survey carried out in 2009–10 by the Centre de Recherche pour l'Etude et l'Observation des Conditions de Vie (CREDOC). The CCAF survey collected data on anthropometry, socio-economic status, lifestyle, and consumption of foods, energy, and macro- and micronutrients in a representative sample of French consumers characterised by age, sex, education level and body adiposity status.

## Methods

### Population

The details of participant recruitment are consistent with the CREDOC methodology, as described in previous publications^(^ ^35^ ^)^. The survey was carried out between October 2009 and July 2010 in a national representative sample of 1222 French households, in which all individuals aged ≥ 3 years were interviewed, plus an extra national sample of 828 individuals aged 3–19 years. Age, socio-economic status, geographical region, town size and household size were taken into consideration in the quota sampling method. For the present analysis, the CCAF 2010 sample was subdivided into three age groups: children (3–12 years, *n* 855); adolescents (13–17 years, *n* 316); adults (18 years and older, *n* 1389).

For each participant, self-reported height and weight and time spent on physical activities and sedentary (screen watching) behaviour were recorded in face-to-face interviews. The participants then completed a 7 d food survey. Data for children were obtained either from the parents (children aged < 9 years) or from the children themselves (aged ≥ 9 years).

The estimated energy intake reported by the included participants was consistent with the estimated energy requirements (1·55 times the metabolic rate), according to Schofield's equation^(^ ^36^ ^)^. Adult participants were excluded if their reported intake was inconsistent with the estimated energy requirements. The proportion of overweight and obese individuals (BMI >25 kg/m^2^) among the excluded participants was greater than that among the included participants (χ^2^; *P*< 0·05). There were no differences in sex distribution and education level between the included and excluded participants.

To control for seasonal differences in intake, the survey was carried out in four successive phases (October–December, January–March, April–mid-June, and mid-June–July), during each of which approximately a quarter of the participants were included.

### Assessment of 7 d food intake

Food intake was assessed on the basis of a 7 d food diary in which details regarding the consumption of all foods and beverages were recorded. The participants were provided with a validated photographic booklet^(^ ^37^ ^)^ showing various common foods and beverages in different portion sizes. Data on the energy and nutrient contents of consumed foods and drinks were obtained from a French food composition table^(^ ^38^ ^)^.

Daily intakes were evaluated for the whole day and for individual eating occasions (main meals and snacks). The circumstances of intake (time of day, day of the week and location) were recorded.

### Assessment of whole grain consumption (amounts and frequency)

The CREDOC food composition table lists thirty-eight food groups based on nutrient content. The CREDOC food groups were checked for the presence of whole grain ingredients (from wheat, oats, barley, rice, maize, rye, buckwheat, quinoa, bulgur, millet, spelt and amaranth). All foods were considered, with no lower limit on whole grain content. Whole grain products were found to be present in the following seven food groups: ‘sweet crackers and biscuits’; ‘breakfast cereals’; ‘bread and toasts’; ‘pasta’; ‘mixed dishes’; ‘rice and cooked cereals’; ‘sweet products’ (including cereal bars and popcorn). Foods in the ‘mixed dishes’ food group containing whole grains were mainly buckwheat pancakes and some specialty dishes (e.g. soya steak with oats). In a few cases, intakes from the ‘sandwiches’ food group contributed to whole grain intake. In these rare cases (*n* 12 events), the whole grain intake was calculated and added to the intake from the ‘bread and toasts’ food group. Minimal whole grain intake (*n* 1) was identified for the ‘cakes and pies’ food group, and this intake was not included in the analysis.

Details regarding the whole grain content of consumed foods were obtained from brand information and quantitative nutrient declarations (QUIDs) on food labels (52 % of all whole grain foods consumed). QUIDs were obtained from MINTEL, a market research database^(^ ^39^ ^)^, manufacturer's websites or online shopping websites. If brand or QUID information was not available, then details of the whole grain content of similar products were used (products of the same brand with available QUIDs (14 % of foods consumed), or similar ingredient list, or similar name and description (34 % of foods)). For ease of analysis and due to the low number of consumers of certain foods, pasta and rice were grouped together, and ‘mixed dishes’, popcorn and cereal bars were merged into one group called ‘Other’.

Total whole grain consumption, computed as the total intake from the various sources mentioned above, was determined in amounts (g/d) and frequency (eating occasions per week) for the total population and for only consumers. Consumers were defined as respondents who reported consumption of a whole grain product at least once in the 7 d food diary. Tertiles of whole grain intake were defined for children and adults. Whole grain intake data were analysed in terms of age, sex, education level, geographical region, physical activity level, smoking status and BMI. The daily distribution of intake was examined in terms of time and place of consumption.

### Determination of body adiposity status

In adults, BMI values between 18·5 and 25 kg/m^2^ were considered to represent normal body adiposity status. Overweight was defined as BMI values between 25 and 30 kg/m^2^ and obesity as BMI values ≥ 30 kg/m^2^. Leanness corresponded to BMI values < 18·5 kg/m^2^. In children, body adiposity status was defined on the basis of growth curves and cut-off values for all age groups presented by Cole *et al.* ^(^ ^40^ ^)^.

### Determination of time spent on physical activities and screen watching

The time spent on watching various screens (television, computer, video games, etc.) as well as the time spent on various physical activities was reported by the participants. For physical activity, two levels were arbitrarily defined in adults (time spent on physical activities such as household activities, gardening, sports, etc.; less or more than 2 h/d) and in children and adolescents (time spent on sports activities; less or more than 4 h/week). For daily screen watching also, two levels were arbitrarily defined in adults (more or less than 3 h/d) and in children and adolescents (more or less than 2 h/d).

### Statistical analyses

The SAS 9.2 software was used for statistical analyses (SAS Institute, Inc.). The means and standard errors for whole grain intake were calculated according to sex, age group, education level, eating occasion, geographical location, and day of the week for the total population and for only consumers. Differences between the proportions were tested using χ^2^ tests. Differences in quantitative variables (such as intakes) were tested using the generalised linear model (PROC GLM) adjusted for energy (ANCOVA) or age and sex (multivariate ANCOVA). Tertile analysis was also carried out according to the levels of whole grain consumption. Multiple comparison tests were carried out with Bonferroni correction.

Multiple logistic regression analysis was carried out to explore eventual associations between whole grain consumption and body adiposity status. The prevalence of obesity in children and in adults was too low to allow any logistic regression; therefore, obesity status was aggregated with overweight. The OR and associated 95 % CI for overweight/obesity for each level of whole grain intake were calculated using multiple logistic regressions, adjusted for age, sex, education level, geographical region, physical activity level (only adults due to missing data in children), smoking status (only adults) and energy intake.

Data are reported as means with their standard errors and/or medians and percentiles. The statistical significance level was set at *P*< 0·05.

In statistical analyses with subsamples of sufficient size, data were analysed separately for children and adolescents. In all other circumstances, data obtained for children and adolescents were combined into one group.

## Results

### Whole grain consumption

The daily intakes of whole grains (g/d) in children (including adolescents) and adults are given in Tables 1 and 2, respectively. The intakes are reported according to age, sex, education level and geographical region for the total population and for only whole grain consumers. Overall, 55 % of the children (*n* 639) and 68 % of the adults (*n* 929) reported that they did not consume whole grains over the 7 d dietary observation period. Among consumers, the mean daily whole grain intake was significantly higher in teenagers aged 13–17 years (12·9 (sem 1·3) g/d) than in younger children aged 3–6 years (6·4 (sem 0·5) g/d) or 7–12 years (8·0 (sem 0·7) g/d) (*P*< 0·001). Among adults, a non-significant trend for higher intakes was observed in older participants with the mean daily intake ranging from 11·2 (sem 0·9) g/d in younger adults to 17·6 (sem 4·3) g/d in adults aged ≥ 75 years (*P*= 0·054).

**Table 1:** Descriptive analysis of whole grain intake (g/d) in French children (total population and only consumers) (Number of children and percentages; mean values with their standard errors; median values and 95th percentiles (P95))

	Total population (*n* 1171)	Only consumers (*n* 532; 45 %)
	*n*	%	Mean	sem	Median	P95	*P* *	*n*	%	Mean	sem	Median	P95	*P* *
All children (3–17 years)	1171	–	4·1	0·3	0·0	20·5		532		9·0	0·5	5·4	26·4	
Age group (years)							0·077							0·001
3–6	354	30	3·0	0,3	0·0	14·4		164	31	6·4^a^	0·5	4·5	19·1	
7–12	501	43	3·7	0·4	0·0	17·9		239	45	8·0^a^	0·7	5·0	22·5	
13–17	316	27	5·3	0·6	0·0	25·9		129	24	12·9^b^	1·3	7·5	39·6	
Sex							0·767							0·614
Boys	596	51	4·1	0·3	0·0	20·6		273	51	9·1	0·6	5·4	25·9	
Girls	575	49	4·0	0·4	0·0	19·1		259	49	9·0	0·8	5·4	26·5	
Education†							0·006							0·068
Below secondary degree‡ (high school)	622	53	3·4^a^	0·3	0·0	18·3		262	49	8·3	0·7	4·6	23·9	
Secondary degree (high school) or above§	549	47	4·7^b^	0·4	0·0	22·2		270	51	9·5	0·7	5·8	26·5	
Geographical region							0·001							0·004
Paris region	188	16	2·5^a^	0·4	0·0	13·3		65	12	7·1^a^	1·0	4·4	22·2	
North	588	50	3·7^a^	0·3	0·0	20·6		275	52	8·0^a^	0·5	5·6	23·8	
South	395	34	5·4^b^	0·6	0·0	21·4		192	36	11·1^b^	1·1	5·4	39·6	
Weekdays *v.* weekend							0·209							0·003
Weekdays	1171		3·9	0·2	0·0	20·7		464	87	10·0^a^	0·5	6·3	30·0	
Weekend	1171		4·4	0·4	0·0	20·6		312	59	16·0^b^	1·3	10·3	45·3	

^a,b^Mean values within a column with unlike superscript letters were significantly different (*P*< 0·05).

* ANCOVA test (ANCOVA test adjusted for energy – Bonferroni *post hoc* test).

† Education refers to household head's education level.

‡ The below secondary degree category includes respondents with no high-school degree.

§ The secondary degree or above category includes respondents with high-school, college, graduate and postgraduate degrees.

**Table 2:** Descriptive analysis of whole grain intake (g/d) in French adults (total population and only consumers) (Number of adults and percentages; mean values with their standard errors; median values and 95th percentiles (P95))

	Total population (*n* 1389)	Only consumers (*n* 460; 32 %)
	*n*	%	Mean	sem	Median	P95	*P* *	*n*	%	Mean	sem	Median	P95	*P* *
All adults (18+ years)	1389	–	4·7	0·3	0·0	26·4		460	–	14·4	0·8	8·1	51·8	
Age group (years)							0·684							0·054
24–34	449	32	4·5	0·5	0·0	25·2		184	40	11·2	0·9	6·6	33·1	
35–54	497	36	5·2	0·7	0·0	30·0		162	35	15·6	1·7	7·8	57·0	
55–74	372	27	4·3	0·6	0·0	26·4		97	21	16·4	1·8	9·9	50·7	
75+	71	5	4·0	1·3	0·0	23·2		17	4	17·6	4·3	13·7	82·0	
Sex							0·001							0·267
Men	588	42	3·9^a^	0·5	0·0	25·4		165	36	13·8	1·3	7·7	51·8	
Women	801	58	5·4^b^	0·5	0·0	27·3		295	64	14·8	1·1	8·3	53·3	
Education†							0·033							0·169
Below secondary degree‡ (high school)	717	52	4·0^a^	0·4	0·0	22·6		185	40	15·9	1·5	8·6	5·6	
Secondary degree (high school) or above§	672	48	5·4^b^	0·5	0·0	30·0		275	60	13·4	1·0	7·5	49·5	
Geographical region							0·001							0·016
Paris region	222	16	5·0^a,b^	0·8	0·0	27·0		79	17	14·2^a,b^	1·9	6·9	49·5	
North	646	47	3·1^a^	0·4	0·0	17·4		185	40	11·6^a^	1·1	7·5	40·3	
South	521	38	6·5^b^	0·7	0·0	39·6		196	43	17·0^b^	1·5	9·7	56·6	
Weekdays *v.* weekend							0·001							0·347
Weekdays	1389		5·0^a^	0·4	0·0	27·8		410		17·2	1·1	9·7	62·5	
Weekend	1389		3·9^b^	0·3	0·0	29·0		256		21·4	1·2	15·0	62·3	

^a,b^Mean values within a column with unlike superscript letters were significantly different (*P*< 0·05).

* ANCOVA test (ANCOVA test adjusted for energy – Bonferroni *post hoc* test).

† Education refers to household head's education level.

‡ The below secondary degree category includes respondents with no high-school degree.

§ The secondary degree or above category includes respondents with high-school, college, graduate and postgraduate degrees.

Among consumers, no significant sex difference was observed in either children or adults. In both children and adults, the mean daily intake of whole grains varied according to geographical region (*P*= 0·001); it was significantly higher in participants from the south of France than in those from the north (11·1 (sem 1·1) *v.* 8·0 (sem 0·5) g/d in children and 17·0 (sem 1·5) *v.* 11·6 (sem 1·1) g/d in adults).

In the total population, the mean daily intake of whole grains was higher in children of more educated parents (high-school degree and above) (4·7 (sem 0·4) g/d) than in those of less educated parents (3·4 (sem 0·3) g/d; *P*= 0·006), but this education-associated difference was no longer significant when considering the mean daily intake in only consumers (9·5 (sem 0·7) *v.* 8·3 (sem 0·7) g/d, respectively; *P*= 0·068). Similarly, in the total adult population, the mean daily intake was higher in participants with higher education (5·4 (sem 0·5) *v.* 4·0 (sem 0·4) g/d; *P*= 0·033), but education level did not significantly affect the mean daily intake among adult whole grain consumers (13·4 (sem 1·0) *v.* 15·9 (sem 1·5) g/d in higher- *v.* lower-education groups, respectively; *P*= 0·169). Among child whole grain consumers, the mean daily intake was higher during the weekends (16 (sem 1·3) g/d) than during the weekdays (10 (sem 0·5) g/d; *P*= 0·003), but there was no significant difference in adults (21·4 (sem 1·2) *v.* 17·2 (sem 1·1) g/d, respectively; *P*= 0·347). Lifestyle variables including physical activity level, screen viewing time and smoking status were not associated with significant differences in whole grain intake (data not shown).

**Fig. 1:**
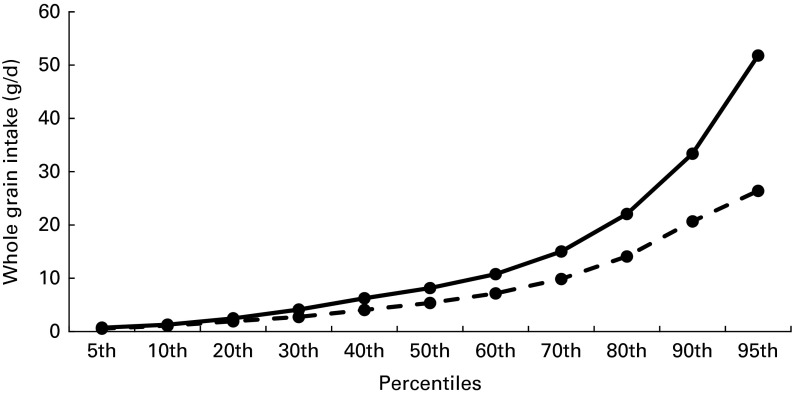
Percentiles of mean daily intake of whole grains in French children (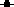, 3–17 years, *n* 532) and adults (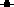, 18+ years, *n* 460) (consumers only).

The percentiles of mean daily intake of whole grains in child and adult consumers are shown in Fig. 1. Whole grain intakes were negatively skewed over half of the consumers' samples, the mean daily intake remaining < 10 g/d. The 95th percentile of intake reached 26 and 52 g/d in children and adults, respectively. The mean number of whole grain eating occasions recorded over 1 week was 1·6 in children and adolescents and 1·2 in adults when the total population was considered. This increased to 3·3, 3·8 and 3·6 eating occasions/week in children, adolescents and adults, respectively, when only consumers were considered.

**Fig. 2:**
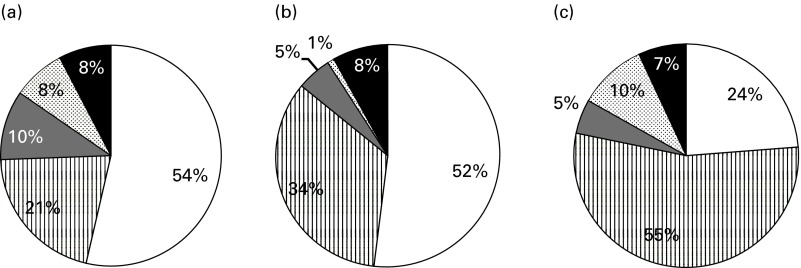
Contribution (%) of different whole grain food groups to total whole grain intake in (a) children (3–12 years, *n* 403), (b) teenagers (13–17 years, *n* 129) and (c) adults (18+ years, *n* 460) (only consumers). 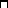, Ready-to-eat breakfast cereals; 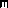, breads and toasts; 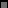, sweet crackers and biscuits; 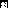, pastas, rice and cooked cereals; 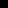, cereal bars, popcorn and other products.

**Fig. 3:**
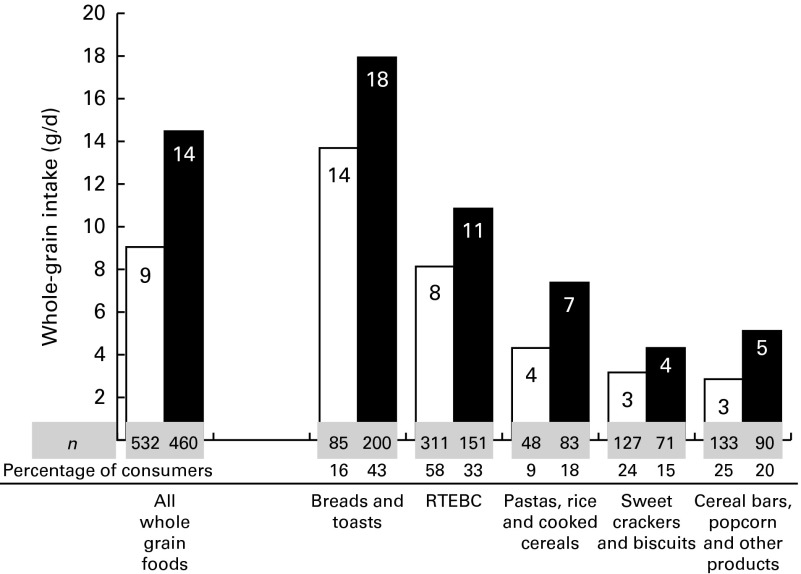
Whole grain intakes (g/d) per food group in children (□, 3–17 years, *n* 532) and adults (■, 18+ years, *n* 460) (in consumers of specific food groups). Percentages of children or adults consuming each food group are indicated below each bar. Data for adolescents are included in the children's sample because of very small sample sizes in some food categories. RTEBC, ready-to-eat breakfast cereals.

The participants consumed whole grains mainly at home (87 % in children and 91 % in adults). In children, over half of the mean daily intake (60 %) was achieved at breakfast and 17 % at dinner. Other minor eating occasions were lunch (9 %) and afternoon snacks (12 %). In adults, the majority of whole grain intake was also achieved at breakfast (47 %), with the remainder being distributed between lunch (20 %) and dinner (26 %). (Complete data describing the distribution of intake at various eating occasions are given in online supplementary Table SA.) The contribution (%) of different food groups to total whole grain intake in children, adolescents and adults is shown in Fig. 2. Ready-to-eat breakfast cereals (RTEBC) accounted for over half of the mean daily intake in children and adolescents, while bread and toasts were the main sources in adults (55 %). (Further details regarding the contribution of different food sources across tertiles of whole grain intake are given in online supplementary Table SB.) In Fig. 3, the whole grain intake (g/d) from various food groups in only consumers of specific food groups is shown. Bread and toasts contributed the highest to the mean daily intake of whole grains in consumers (14 g/d in 16 % of child consumers and 18 g/d in 43 % of adult consumers). Sweet crackers and biscuits, on the other hand, provided only 3 and 4 g/d in child consumers (24 %) and adult consumers (15 %), respectively. Wheat was the major source of whole grains in the diet, accounting for 72, 76 and 64 % of the intake in children, adolescents and adults, respectively. Other sources were oats (8–15 %) and buckwheat (7–9 %).

### Diet composition of consumers and non-consumers of whole grains

The intakes of energy and macro- and micronutrients in non-consumers *v.* consumers of whole grains across tertiles of whole grain intake in children (including adolescents) and adults are given in Table 3. Total energy intake did not differ significantly between consumption levels, but significant differences were observed for several nutrients. In children and adults, the mean daily intakes of fibre, vitamins B_1_, B_2_, B_3_, B_5_, B_6_, B_9_, C, and E, Ca, Fe, Mg and Mn tended to be higher as the daily intake of whole grains increased. In only adults, the mean daily intakes of β-carotene, K and Cu were also significantly higher as the levels of whole grain consumption increased. Among children and adults, there was a significantly lower intake of simple sugars in the diet of non-consumers than in consumers. There were no significant differences in fat intake.

**Table 3:** Mean daily intakes of energy including alcohol (MJ), macronutrients (% of total non-alcohol energy intake (NAEI)) and micronutrients (g, mg, or μg/10 MJ) in French child and adult non-consumers of whole grains and across tertiles of whole grain intake (WGI)

	WGI (g/d)
	Children (3–17 years, *n* 1171)	Adults (18 years and above, *n* 1389)
	WGI = 0 (*n* 639)	0 < WGI < 3 (*n* 186)	3 ≤ WGI < 8·9 (*n* 190)	WGI ≥ 8·9 (*n* 156)	*P* *	WGI = 0 (*n* 929)	0 < WGI < 4·4 (*n* 157)	4·4 ≤ WGI < 13·3 (*n* 158)	WGI ≥ 13·3 (*n* 145)	*P* *
Energy (MJ)	7·6	7·2	7·5	8·1	NS	8·9	9·0	9·3	9·4	NS
Carbohydrates (%g/NAEI)	60·3	60·9	60·5	61·1	NS	56·9^a^	58·9^b^	58·2^a,b^	58·1^a,b^	< 0·01
Simple sugars (%g/NAEI)	28·6^a^	31·4^b^	30·6^b^	30·4^b^	< 0·001	22·1^a^	26·1^b^	24·9^b^	25·5^b^	< 0·001
Proteins (%g/NAEI)	20·9	20·1	20·6	20·6	NS	22·8^a^	21·1^b^	21·9^a,b^	21·6^b^	< 0·001
Fat (%g/NAEI)	18·8	19·0	18·9	18·3	NS	20·3	20·0	19·9	20·3	NS
SFA (%g/NAEI)	8·1	8·3	8·4	8·0	NS	8·6	8·3	8·5	8·6	NS
MUFA (%g/NAEI)	6·6	6·5	6·5	6·3	NS	7·0	7·0	6·8	7·1	NS
PUFA (%g/NAEI)	2·3	2·4	2·3	2·3	NS	2·9	2·9	2·9	3·0	NS
Fibre (g/10 MJ)	18·5^a^	18·1^a^	18·3^a,b^	19·5^b^	< 0·01	19·4^a^	19·7^a,b^	21·4^b^	23·8	< 0·001
Cholesterol (mg/10 MJ)	390·7	372·6	383·9	385·0	NS	416·1^a^	397·1^a,b^	412·1^a,b^	377·1^b^	< 0·01
Vitamin A (μg/10 MJ)	1046·6	1040·8	1054·5	1161·7	NS	1487·4	1388·0	1479·1	1468·9	NS
β-Carotene (μg/10 MJ)	2976·6	2795·2	2798·3	3151·2	NS	3663·6^a^	4248·7^a,b^	3991·9^a,b^	4123·9^b^	< 0·001
Retinol (μg/10 MJ)	550·5	574·9	588·1	636·5	NS	876·8	679·9	813·8	781·6	NS
Vitamin B_1_ (mg/10 MJ)	1·6^a^	1·6^a^	1·6^a^	1·9^b^	< 0·001	1·4^a^	1·5^b^	1·5^b^	1·7	< 0·001
Vitamin B_2_ (mg/10 MJ)	2·2^a^	2·3^a^	2·3^a^	2·6^b^	< 0·001	2·1^a^	2·2^a^	2·1^a^	2·3^b^	< 0·001
Vitamin B_3_ (mg/10 MJ)	20·4^a^	19·5^a^	20·2^a^	24·2^b^	< 0·001	20·9^a^	20·5^a^	20·8^a^	23·2^b^	< 0·001
Vitamin B_5_ (mg/10 MJ)	6·7^a^	6·6^a^	6·7^a^	8·1^b^	< 0·001	6·2^a^	6·3^a^	6·4^a^	7·0^b^	< 0·001
Vitamin B_6_ (mg/10 MJ)	2·2^a^	2·2^a^	2·2^a^	2·6^b^	< 0·001	2·2^a^	2·2^a^	2·3^a^	2·5^b^	< 0·001
Vitamin B_9_ (μg/10 MJ)	310·8^a^	324·8^a^	327·7^a^	365·4^b^	< 0·001	309·0^a^	330·6^b^	343·9^b^	379·7	< 0·001
Vitamin B_12_ (μg/10 MJ)	5·8	6·0	5·4	6·4	NS	7·3	6·6	6·8	7·0	NS
Vitamin C (mg/10 MJ)	109·2^a^	117·8^a,b^	125·3^b^	124·0^b^	< 0·01	95·9^a^	117·4^b,c^	111·3^b^	131·3	< 0·001
Vitamin D (μg/10 MJ)	2·5	2·6	2·5	2·4	NS	2·8	2·6	2·9	3·1	NS
Vitamin E (mg/10 MJ)	9·0^a^	10·1^b^	9·2^a,b^	9·7^b^	< 0·001	9·1^a^	10·0^a,b^	10·0^b^	10·8^b^	< 0·001
Ca (mg/10 MJ)	1138·8^a^	1205·1^a,b^	1191·0^a,b^	1267·9^b^	< 0·001	980·2^a^	1009·0^a,b^	1059·9^b^	1071·4^b^	< 0·001
Fe (mg/10 MJ)	14·8^a^	14·8^a^	15·0^a^	16·7^b^	< 0·001	15·0^a^	15·1^a^	15·0^a^	17·5^b^	< 0·001
Zn (mg/10 MJ)	10·8	10·6	10·7	11·0	NS	11·5	10·9	11·3	11·3	NS
Na (mg/10 MJ)	3176·5^a^	3021·5^b^	3033·9^b^	3068·8^a,b^	< 0·05	3649·9	3460·6	3556·2	3480·4	NS
I (μg/10 MJ)	141·9	146·5	141·3	148·2	NS	133·6	137·9	141·5	139·7	NS
Mg (mg/10 MJ)	303·8^a^	304·4^a,b^	304·5^a,b^	315·2^b^	< 0·01	313·4^a^	316·1^a^	323·7^a^	357·4^b^	< 0·001
Mn (mg/10 MJ)	2·6^a^	2·7^a,b^	2·7^a,b^	2·8^b^	< 0·001	2·9^a^	3·3^b^	3·3^b^	4·1	< 0·001
P (mg/10 MJ)	1610·0	1569·7	1582·7	1618·5	NS	1527·2	1488·0	1528·7	1553·9	NS
K (mg/10 MJ)	3393·3	3398·2	3368·1	3473·6	NS	3325·4^a^	3374·6^a^	3447·1^a,b^	3591·4^b^	< 0·001
Se (μg/10 MJ)	58·0	55·1	58·3	57·7	NS	62·1	60·1	63·8	64·2	NS
Cu (mg/10 MJ)	1·6	1·6	1·6	1·7	NS	1·6^a^	1·6^a^	1·7^a,b^	2·0^b^	< 0·001

^a,b,c^Mean values within a row with unlike superscript letters were significantly different (*P*< 0.05).

* Multivariate ANCOVA test (ANCOVA test adjusted for age and sex – Bonferroni *post hoc* test).

Food selection also varied between consumers and non-consumers of whole grains. Child and adult consumers of whole grains consumed significantly more dairy products and vegetables when compared with non-consumers. Adult consumers of whole grains also consumed significantly more fruits and fish, but less meat and potatoes when compared with non-consumers. (Complete data for consumption of food groups and for whole grain intakes from food groups in children and adults are given in online supplementary Tables SB and SC).

### BMI in consumers and non-consumers of whole grains

The prevalence of overweight and obesity combined in non-consumers *v.* consumers of whole grains across tertiles of whole grain intake in children and adults is summarised in Table 4. The OR and 95 % CI for overweight/obesity are also given. A logistic regression analysis with adjustment for age, sex, education level, geographical region, physical activity level (only adults), smoking status (only adults) and energy intake revealed a significant trend in adults. The OR for being overweight or obese in adult non-consumers of whole grains was 1·7 (95 % CI 1·1 ≤ OR ≤ 2·5, *P*= 0·044) when compared with adult consumers in the highest tertile. Further adjustments for intakes of fruits, vegetables and dairy products did not change the significance of the effects.

**Table 4:** Prevalence of overweight and obesity in non-consumers and across tertiles of whole grain intake (WGI) (Number of participants and percentages; odds ratios and 95 % confidence intervals)

	WGI (g/d)
	Children (3–17 years, *n* 1171)	Adults† (18 years and above, *n* 1389)
Overweight/obese‡	WGI = 0 (*n* 639)	0 < WGI < 3·2 (*n* 186)	3·2 ≤ WGI < 9·9 (*n* 190)	WGI ≥ 9·9 (*n* 156)	*P* for trend	WGI = 0 (*n* 929)	0 < WGI < 4·9 (*n* 157)	4·9 ≤ WGI < 15·6 (*n* 158)	WGI ≥ 15·6 (*n* 145)	*P* for trend
Prevalence
*n*	121	26	30	26		420	50	55	45	
%	18	14	16	15		47	34	38	33	
OR	1·1	0·8	0·9	1·0	0·3969	1·7*	1·4	1·3	1·0	0·0439
95 % CI	0·7, 1·8	0·4, 1·5	0·5, 1·6	Reference		1·1, 2·5	0·8, 2·3	0·8, 2·1	Reference	

* Value is significantly different from that of adult consumers in the highest tertile (*P*< 0·05).

† In adults, difference remained statistically significant after further adjustments for fruit, vegetable and dairy product intakes.

‡ Logistic regression on ‘being overweight or obese’ – reference third tertile – adjusted for age, sex, education level, geographical region, physical activity level (only adults), smoking status (only adults) and energy intake.

### Whole grain consumption and dietary recommendations

The French Nutrition and Health Program^(^ ^31^ ^)^ recommends that one serving of starchy food be consumed with every meal, preferably ‘whole food’. The 7 d food records indicated that the mean daily numbers of servings of starchy foods (grain products, potatoes and legumes) consumed by children, adolescents and adults were 2·6, 3·5 and 3·7, respectively. These numbers of servings roughly matched the recommended frequency of intake. However, the frequency of selecting whole grain options was low for ‘bread and toasts’ and ‘pasta, rice and cooked cereals’ (about 2–4 % of all servings). Higher proportions of RTEBC servings contained whole grains (34, 46 and 61 % in children, adolescents and adults, respectively).

Very low percentages of adolescent and adult whole grain consumers achieved the American recommendation of 48 g of whole grains/d (3 and 7 %, respectively), and no child achieved this level of intake. The proportion of participants meeting the estimated average requirements^(^ ^41^ ^)^ for various nutrients was compared between consumers and non-consumers of whole grains. A significantly higher proportion of whole grain consumers met the estimated average requirements for vitamins A, B_9_, C, and E, Ca, Fe, Zn, I and Mg (children and adults) and for vitamins B_5_ and B_6_, Se, and Cu among only adults (online supplementary Table SD).

## Discussion

The present study is the first to specifically assess whole grain intake in a representative sample of the French population. The results reveal that children, adolescents and adults in France consume very low amounts of whole grains. Less than half of the respondents in all age groups reported consuming any whole grain at all over 7 d. Among consumers, the mean daily intake was 6–8 g in children, 13 g in adolescents and 14 g in adults, and they consumed whole grains only three to four times per week. The 2006 ‘Etude Nationale Nutrition Santé’ survey^(^ ^32^ ^)^, which aimed to assess the compliance with the national dietary recommendations established by the PNNS, revealed that most French consumers indeed consumed one or more starchy foods with each meal as recommended (average 3·7 servings/d in adults), in agreement with the observations made in the present study. However, 55 % of the adults and 62 % of the children did not report any intake of whole grain products in the 3 d food records. The ongoing internet-based Nutrinet-Santé Study has collected food intake data from 148 962 French volunteers^(^ ^34^ ^)^. In 2011, it reported that only 16 % of the participants consumed at least one whole grain food serving out of three daily starchy food servings (press release, 11 November 2011, http://www.etude-nutrinet-sante.fr). Consistent evidence indicates that French consumers of all age groups comply with the PNNS recommendation regarding the frequency of starchy food consumption, but this is achieved with a minimal intake of whole grain cereals.

Reported intakes were much below the various quantitative recommendations proposed in developed countries (e.g. 75 g/d in Denmark^(^ ^19^ ^)^ and 48 g in the USA^(^ ^20^ ^)^). They were also much below the reported intakes in other populations. For example, in the USA, mean daily intakes in children are 9·4–14·4 g/d, mainly from breakfast cereals and bread^(^ ^24^ ^,^ ^25^ ^)^. In the UK, Ireland and Germany, mean daily intakes in children are 13·0, 18·6 and 24·3 g/d, respectively, with bread and breakfast cereals being the main contributors^(^ ^23^ ^,^ ^26^ ^,^ ^42^ ^)^. In adults, mean daily intakes in the USA are about 10–12 g/d^(^ ^4^ ^)^, while they are 23 g/d in the UK^(^ ^27^ ^)^, 25–33 g/d in Ireland^(^ ^28^ ^)^, 41–58 g/d in Sweden^(^ ^29^ ^)^ and 37–55 g/d in Denmark^(^ ^29^ ^,^ ^30^ ^)^. As observed in the present study, the major sources of whole grains in other European and US children and adult populations are bread and breakfast cereals.

The reasons for such low levels of whole grain consumption in France are unclear. The total consumption of grain-based products was about 250 and 300 g daily in children and adults, respectively (online supplementary Table SB). Very few of these food choices were from whole grain options. French consumers apparently enjoy bread, pasta and rice, among other sources of grain, but in their refined forms rather than as whole grain products. ‘Breakfast cereals’ was the only food group that French consumers of all age groups were inclined to consume frequently in whole grain versions (from 34 to 61 % of breakfast cereals according to age). In American and Irish populations, barriers to the consumption of whole grains include taste, insufficient awareness of the health benefits or recommendations, lack of preparation skills, perceived cost, and family influences, particularly in families with young children^(^ ^43^ ^,^ ^44^ ^)^. The same factors could similarly affect French consumers, but this should be confirmed by further investigation.

The absence of a specific quantified recommendation for whole grain intake in the PNNS^(^ ^31^ ^)^ may also be a contributing factor. The PNNS recommends that one starchy food be consumed with each main meal, preferably whole food, but leaves portion size to be determined by the consumer's appetite. Although preference for whole food options is explicitly recommended, no quantitative advice is given. Consequently, a consumer can fully comply with the French PNNS recommendations while never consuming whole grain products. The plan also recommends consuming five fruits and vegetables a day and provides definitions of adequate portion sizes for fruits and vegetables. It also recommends that at least three dairy products be consumed daily. These two recommendations are followed by 46 and 29 % of the French adult population, respectively (press release, November 11 2011, http://www.etude-nutrinet-sante.fr). Compliance with these recommendations is higher than for whole grains, which might be because the recommendations are more explicit in terms of the number and size of servings.

In Denmark, the consumption of whole grains has increased over recent years from 32 g/d in 2000–4 to 55 g/d in 2011–2^(^ ^30^ ^)^. This change occurred following a national campaign enforced with the purpose of increasing the consumption of whole grains in Denmark. The campaign included different approaches: increasing whole grain content in a number of commercial food products; communication to improve consumers' knowledge about whole grain foods and their health benefits; use of a specific whole grain logo on foods with a high content of whole grains ( ≥ 60 % per dry weight) that also met strict criteria for total sugar ( ≤ 13 %), fat ( ≤ 7 %) and Na ( ≤ 500 mg/100 g) contents (http://www.fuldkorn.dk/media/104749/Logo.manual.english.may2013.pdf). This campaign resulted in clear increases in daily consumption of whole grains in children and adults, in males and females, and in increased proportions of the population meeting the Danish recommendation (75 g whole grain/10 mJ per d). Similar strategies that address documented barriers to whole grain consumption^(^ ^43^ ^,^ ^44^ ^)^ could be adopted in France to increase the consumption of whole grains above the minimal levels observed in all segments of the population.

Despite the extremely low levels of consumption in most participants, it appeared that consumers of whole grains, and in particular those in the highest tertile of intake ( ≥ 9 g/d), ate a higher-quality diet when compared with non-consumers. Fibre and many micronutrients including B vitamins, Fe and Ca were consumed in higher amounts by whole grain consumers. These observations are in line with those of American and European studies^(^ ^4^ ^23^ ^45^ ^–^ ^47^ ^)^, in which the levels of whole grain consumption were considerably higher. Furthermore, higher percentages of whole grain consumers than non-consumers achieved daily intake levels that matched the estimated average requirements^(^ ^41^ ^)^ for a number of vitamins and minerals. Whole grain products alone contributed about 7–10 % of total fibre in the diet of child and adult whole grain consumers. As only 22 % of French men and 12 % of French women currently meet the 25 g minimum recommended fibre intake levels (press release, November 22 2012, http://www.etude-nutrinet-sante.fr), there appears to be a high potential to improve fibre intake in the French population through increased consumption of whole grain foods. Whole wheat flour, for example, contains more fibre (11 *v.* 3·9 g/100 g) than refined wheat flour and also more vitamins and minerals^(^ ^38^ ^)^, which suggests an opportunity for improving the intake of micronutrients.

Notably, whole grain consumers in the present study had a higher daily intake of simple sugars than non-consumers, despite similar total energy intakes. As RTEBC were the main source of whole grains in children's diets, it is possible that the higher total sugar intakes observed in whole grain consumers were due to higher intakes of added sugars from RTEBC, and also higher intakes of lactose. Examination of the sources of simple sugars in the diet of child whole grain consumers revealed that beverages contributed 28 % of the daily sugar intakes and all dairy products 21 %, while all whole grain products (including RTEBC) contributed 5 %. In adult whole grain consumers, the higher total sugar intakes may be related to the consumption of fresh and dried fruits, dairy products and whole grain products as intakes of these food groups were all significantly higher in them. As done in the successful Danish campaign implemented to increase the consumption of whole grain foods^(^ ^30^ ^)^, consideration should be given to the overall nutrient profile of recommended whole grain sources.

The prevalence of overweight and obesity observed in children and adults in the present study was consistent with other reports based on measured rather than self-reported body height and weight values. A cross-sectional study conducted in French children in 2007 showed the prevalence of overweight to be 15·8 % and that of obesity to be 2·8 % ^(^ ^48^ ^)^. In French adults, recent prevalence values for overweight and obesity based on measured height and weight are 25·6 and 10·6 %, respectively^(^ ^49^ ^)^. These data are consistent with the low prevalence of obesity observed in the present study. However, given the small number of obese children and adults in the present study, logistic regression could not be computed to assess the risk of obesity *per se*. Instead, obesity was aggregated with overweight in a logistic regression analysis adjusted for several factors known or suspected to affect body adiposity status (age, sex, education level, geographical region, physical activity level, smoking status and total energy intake). A significant trend was found in adults, but not in children, showing a decreasing OR of being overweight or obese with increasing levels of whole grain consumption. Further adjustments for intakes of fruits, vegetables and dairy products did not modify the results. The absence of significant effects in children may be due to the overall low level of whole grain consumption even in consumers and the shorter exposure to whole grains in young people than in adults. The cross-sectional nature of the study prevents the identification of causal links, but the trend observed in adults suggests that whole grain intake might be an important marker of a lifestyle favouring adequate body weight control. This hypothesis is consistent with previous epidemiological reports of a beneficial effect of whole grain consumption on body weight control in children and adults^(^ ^4^ ^,^ ^5^ ^,^ ^45^ ^)^.

The strengths of the present study are the representative nature of the population, covering all age groups, and the use a validated 7 d food record that provided a very complete picture of dietary intake in France. Further strengths were the comprehensive approach taken to estimate whole grain intake from all dietary sources and the use of brand information to define the whole grain content of over 50 % of the reported whole grain products consumed. Nevertheless, a certain level of uncertainty remains in the assessment of whole grain content of many products on the basis of food labels and/or websites. Under-reporting remains a possibility in this population, although energy intake seemed to be compatible with body energy requirements. The exclusion of potential participants with reported energy intake levels below the estimated energy requirements, among which the proportion of overweight/obese was greater than that among the participants included, prevented the analysis of whole grain consumption in a fraction of the population. Consequently, whole grain consumption may have been mis- or underestimated. The cross-sectional nature of the study precluded the observation of causal effects. Although whole grain consumption is associated with a variety of individual and lifestyle factors, as well as a higher intake of a variety of nutrients, the potential causal links between these observations remain unclear. Whole grain consumption could be a marker of and/or a significant contributor to a healthy lifestyle facilitating good nutrition and weight status.

In conclusion, very low levels of whole grain consumption are observed in every age group in the French population. Most of the respondents reported never consuming any whole grains, and consumption levels were extremely low among those who did. The main sources of whole grain intake in the French diet are breakfast cereals, bread and toasts. In spite of these very low consumption levels, consumption of whole grain products is associated with higher intakes of fibre and some vitamins and minerals. Although the French PNNS recommends consuming one starchy food with each main meal, preferably a whole food option, there seems to be only minimal preference for whole grain foods in the daily diet of most French consumers. The risk of being overweight or obese is reduced in adult whole grain consumers. Increasing the consumption of whole grains in the French population seems an important opportunity for improving overall dietary health, in particular, intakes of dietary fibre, which are currently low. However, to achieve this goal, the barriers to whole grain consumption in French adults and children must first be addressed.

## Supplementary material

To view supplementary material for this article, please visit http://dx.doi.org/10.1017/S0007114514002670
